# Pharmaceutical Cocrystal of Piroxicam: Design, Formulation and Evaluation

**DOI:** 10.15171/apb.2017.048

**Published:** 2017-09-25

**Authors:** Prabhakar Panzade, Giridhar Shendarkar, Sarfaraj Shaikh, Pavan Balmukund Rathi

**Affiliations:** ^1^Department of Pharmacognosy, Nanded Pharmacy College, Opp. Kasturba Matruseva Kendra, Shyam Nagar, Nanded, India.; ^2^Department of Pharmaceutics, Shri Bhagwan College of Pharmacy, Dr. Y. S. khedkar Marg, CIDCO, Aurangabad, India.

**Keywords:** Cocrystal, Dissolution, Factorial design, Orodispersible tablet, Piroxicam, Solubility

## Abstract

***Purpose:*** Cocrystallisation of drug with coformers is a promising approach to alter the solid sate properties of drug substances like solubility and dissolution. The objective of the present work was to prepare, formulate and evaluate the piroxicam cocrystal by screening various coformers.

***Methods:*** Cocrystals of piroxicam were prepared by dry grinding method. The melting point and solubility of crystalline phase was determined. The potential cocrystal was characterized by DSC, IR, XRPD. Other pharmaceutical properties like solubility and dissolution rate were also evaluated. Orodispersible tablets of piroxicam cocrystal were formulated, optimized and evaluated using 3^2^ factorial design.

***Results:*** Cocrystals of piroxicam-sodium acetate revealed the variation in melting points and solubility. The cocrystals were obtained in 1:1 ratio with sodium acetate. The analysis of Infrared explicitly indicated the shifting of characteristic bands of piroxicam. The X-Ray Powder Diffraction pattern denoted the crystallinity of cocrystals and noteworthy difference in 2θ value of intense peaks. Differential scanning calorimetry spectra of cocrystals indicated altered endotherms corresponding to melting point. The pH solubility profile of piroxicam showed sigmoidal curve, which authenticated the pKa-dependent solubility. Piroxicam cocrystals also exhibited a similar pH-solubility profile. The cocrystals exhibited faster dissolution rate owing to cocrystallization as evident from 30% increase in the extent of dissolution. The orodispersible tablets of piroxicam cocrystals were successfully prepared by direct compression method using crosscarmelose sodium as superdisintegrant with improved disintegration time (30 sec) and dissolution rate.

***Conclusion:*** The piroxicam cocrystal with modified properties was prepared with sodium acetate and formulated as orodispersible tablets having faster disintegration and greater dissolution rate.

## Introduction

The solubility and dissolution rate of drugs is a decisive factor after oral administration for rate and extent of absorption. This factor offers key challenge for the development and formulation of effective drug in the pharmaceutical industry. More than 60% drugs coming from synthesis and 40% drugs in the development are poorly soluble and face bioavailability problems. Various strategies have been well documented to enhance solubility and dissolution of poorly soluble drugs *viz* salt formation, solid dispersion, microemulsification, cosolvency, inclusion complex formation with cyclodextrin etc.^1-4^

Pharmaceutical cocrystal is a budding tool to modify solubility, dissolution rate and physical and chemical stability of drug substances while keeping the pharmacological effect of drug unchanged. Cocrystal can be defined as stoichiometric multi-component system connected by non-covalent interactions in which two distinct components are solid under ambient conditions. A pharmaceutical cocrystal constitutes active pharmaceutical ingredient and benign substance called a coformer. The cocrystals of piroxicam were reported with different carboxylic acid by solution crystallization, melt crystallization and solvent drop grinding method.^5-7^

Piroxicam is nonsteroidal anti-inflammatory BCS class II drug with prevalent solubility problem. It takes about 3-5 hrs to reach peak plasma concentration after oral administration. This indicates poor absorption of piroxicam after oral administration. Drug dissolution in biological fluid is slow due to limited aqueous solubility leading to erratic bioavailability and suboptimal efficacy. Drug dissolution *in vivo* is the rate-controlling step in drug absorption. It is indicated for acute or long-term use in the relief of signs and symptoms of osteoarthritis and rheumatoid arthritis.^8-12^

Rapid onset and improved bioavailability are desirable for analgesics. Hence there is strong scientific and clinical need to prepare novel forms of piroxicam possessing modified solubility and dissolution rate which can be formulated for oral administration. Accordingly aim of the present study was to prepare pharmaceutical cocrystal of piroxicam, formulation of orodispersible tablets containing piroxicam cocrystal and its evaluation.^13-20^

## Materials and Methods

Piroxicam was gift sample from the Shreya Life sciences Aurangabad (India). All other chemicals were purchased from the SD Fine Chemicals Mumbai (India). Double distilled water was used throughout the research.

### Preparation of cocrystal

Dry grinding method was employed for the preparation of piroxicam cocrystals. Drug and coformer were mixed in different molar ratio (1:1 and 1:2) in mortar and pestle for 45 min to form cocrystals. This was dried an overnight at ambient temperature and stored in tight containers. The 20 coformers screened were adipic acid, benzoic acid, cinnamic acid, citric acid, glutaric acid, p-hydroxybenzoic acid, hippuric acid, malonic acid,resorcinol, saccharine sodium, 1-hydroxy-2-napthoic acid, sodium acetate, urea, catechol, ferulic acid, aerosil-200, nicotinamide, para amino benzoic acid, anthranilic acid and succinic acid.^21,22^

### Determination of melting point 

Melting point of the compounds were estimated using digital melting point apparatus.

### Saturation solubility

The solubility was determined by dissolving excess quantity of pure drug and cocrystals in the 10 ml vials containing water. The vials were subjected to agitation on rotary shaker and allowed to stand for equilibrations for 24 hrs. The samples were filtered after 24 hrs, diluted with distilled water and analyzed by UV Spectrophotometer at 353 nm.^23^

### IR spectroscopy

IR spectroscopy was employed to determine the probable interaction between drug and coformer. The samples were dispersed in KBr pellet and scanned using Shimadzu IR Spectrophotometer between 4000-400 cm^-1^ with resolution of 4 cm^-1^.

### Differential scanning calorimetry

The thermal behavior of drug alone and cocrystal was determined by Differential scanning calorimetry (DSC) studies by Mettler Toledo DSC 822e Module. Weighed samples were heated in aluminum pans at a rate of 5 °C/min, from 0 to 300 °C temperature range, under a nitrogen stream. The instrument was calibrated using indium and empty aluminum pan was used as a reference.

### Powder X-ray diffraction

The silicon sample holders were utilized to get diffraction patterns of pure Piroxicam and cocrystal (Bruker D8 Advance Diffractometer). The instrument was equipped with a fine focus X-ray tube and each sample was placed on to a goniometer head that was motorized to permit spinning of the sample during data acquisition.

### Effect of pH on solubility of piroxicam

The solubility of piroxicam was determined in the various buffers, pH 1 to pH 10 individually. Excess amount of piroxicam was added in the vials containing 10 ml of each buffer. The vials were subjected to rotary shaking and allowed to stand for equilibrations for 24 hrs. The samples were filtered after 24 hrs, diluted with distilled water and analyzed by UV Spectrophotometer at 353 nm.^24^

### Effect of pH on solubility of piroxicam cocrystal

The piroxicam cocrystals in excess quantity were dissolved in hydrochloric acid buffer (pH 1.2), acetate buffer (pH 4.5) Phosphate buffer (pH 6.8 and pH 7.4). The vials were subjected to agitation on rotary shaker and allowed to stand for equilibrations for 24 hrs. The samples were filtered after 24 hrs, diluted with distilled water and analysed by UV Spectrophotometer at 353 nm.^25^

### Powder dissolution study

Dissolution studies were performed in 0.1 N HCl (900 ml) for 60 min at 37±0.5°C and 50 rpm using USP type II dissolution test apparatus (Electrolab, Mumbai, India). The pure drug and cocrystal equivalent to 20 mg of drug was used for the study. The 5 ml of samples were withdrawn after specified time interval and analyzed by UV spectrophotometer at 353 nm.

### Formulation of orodispersible tablets of piroxicam cocrystal by 3^2^ full factorial design

An accurately weighed quantity of piroxicam cocrystal equivalent to drug dose and all other ingredients were passed through 60-mesh sieve and mixed in vertical blender for 30 min. The resulting blend was directly compressed into tablets. The quantity of all components was constant except superdisintegrant and binder. Round concave tablets of 200 mg in weight and 4 mm in diameter were prepared using Cadmach multi station tablet compression machine. Table 1 outlines the composition of various orodispersible tablet formulations.

### Evaluation of pre-compression parameters 

Prior to compression, powder blends were evaluated for tapped density, bulk density, and flow and compressibility parameters. Flow properties of powder were determined by angle of repose and compressibility by Carr's index and Hausner ratio.

**Table 1:** Composition of factorial design formulations

**Ingredients**	**F1**	**F2**	**F3**	**F4**	**F5**	**F6**	**F7**	**F8**	**F9**
Piroxicam cocrystal	24.96	24.96	24.96	24.96	24.96	24.96	24.96	24.96	24.96
Crosscarmillose sodium	10	7	10	4	7	4	10	7	4
MCC PH102	103.04	100.04	100.04	109.04	106.04	106.04	97.04	110.04	103.04
Mannitol	54	54	54	54	54	54	54	54	54
PVP K-30	4	10	7	4	4	7	10	7	10
Aspartame	2	2	2	2	2	2	2	2	2
Magnesium Stereate	2	2	2	2	2	2	2	2	2
Total	200	200	200	200	200	200	200	200	200

### Evaluation of post compression parameters 

#### Thickness and weight variation

The thickness of the tablets was measured using a digital Vernier caliper. Five tablets were randomly taken from each formulation and thickness of each of these tablets was measured. The results are expressed mean±standard deviation (SD). Twenty tablets were selected at random and average weight was determined using an electronic balance (Shimadzu). Tablets were weighed individually and compared with average weight.

#### Hardness and friability

Five tablets were randomly selected from each batch and hardness of tablets was determined by using Monsanto hardness tester. The mean values and standard deviation for each batch were calculated. The friability of tablets was measured using USP type Roche friabilator. Preweighed tablets (equivalent to 6.5 g) were placed in plastic chambered friabilator attached to motor revolving at a speed of 25 rpm for 4 min. The tablets were then dedusted, reweighed, and percent weight loss was calculated using the formula, % friability = ((initial weight–final weight)/ initial weight)×100.

#### Wetting time

Six circular tissue papers of 10 cm diameter were placed in a Petri dish and 10 ml of water containing amaranth dye was added to it to identify complete wetting of tablet surface. A tablet was carefully placed on the surface of tissue paper in Petri dish at ambient temperature. The time taken by water to reach upper surface of the tablet and to completely wet the tablet was noted as wetting time. The study was performed in triplicate and time was recorded using stopwatch.

#### In vitro disintegration time

The digital tablet disintegration test apparatus (Veego) was used to determine *in vitro* disintegration time (DT) using distilled water at 37±2°. The time in seconds taken by tablet for complete disintegration with no residue remaining in apparatus was recorded as mean±SD.

#### In vitro drug release study

The drug release studies were performed using the USP dissolution test apparatus (VDA-6DR USP Stds.,Veego) employing paddle method. The dissolution test was performed using 900 ml of 0.1 N hydrochloric acid at 37±0.5° and paddle speed of 50 rpm. Samples (5 ml) were collected at predetermined time intervals (5 min) and replaced with equal volume of fresh medium. The study was continued for 60 min, samples were then filtered through 0.45 μm membrane filter and analyzed at 353 nm using UV spectrophotometer (Shimadzu).

#### Water Absorption Ratio

A piece of tissue paper folded twice was placed in small Petri dish (7.5cm) containing 7 ml water. A tablet was put on the tissue paper and allowed to wet completely. The wetted tablet was then weighed. The water absorption ratio R was determined using following equation R=W_a_-W_b_/W_a_×100

#### Drug content

Twenty tablets were weighed and powdered. Powder equivalent to a single dose of piroxicam was weighed, dissolved in few ml of methanol, diluted with 0.1N hydrochloric acid and assayed for drug content at 353 nm using UV-Visible spectrophotometer (Shimadzu).

#### Stability study

The optimized formulation was subjected to stability study according to ICH guidelines, at room temperature, 30±2°/60%RH±5% and 40±2°/75% RH±5% condition in stability chamber (HMG, India) for three months. Tablets were assayed for drug content for 90 days at the interval of one month.^26,27^

Preliminary trial formulations of piroxicam cocrystal were framed by direct compression method using varying concentration of superdisintegrant (crosscarmellose sodium) and binder (PVP K-30). The 3^2^ factorial design was used for the optimization of variables (Design Expert 8.0.7.1). The two independent factors, concentration of crosscarmelose sodium (X1) and concentration of PVP K-30 (X2), were set to three different levels and experimental trials were performed for all nine possible combinations. The dependent responses measured were *in vitro* disintegration time (Y1) and percent drug release (Y2).

## Results and Discussion

The 20 coformers were screened for potential cocrystal formation with piroxicam by dry grinding method. Only sodium acetate successfully interacted with piroxicam, giving novel cocrystal form. The obtained piroxicam cocrystal was subjected to physichochemical evaluation and orodispersible tablet formulation.

### Melting point and saturation solubility

The melting points of pure drug, coformers and cocrystals were determined and recorded in Table 2. The saturation solubility of pure drug and potential cocrystals were also determined and reported in Table 2. Both these parameters were estimated as a preliminary screen for potential cocrystals. Melting points of cocrystals were lesser than the piroxicam. The depression of melting points revealed multi component system and designated formation of cocrystals. The modified melting points of cocrystals might be attributed to the interaction between piroxicam and coformers, change in crystallinity of molecules or different packing arrangement. This interaction results in some change in molecular arrangement leading to new crystal form possessing modified physical properties *viz.* melting point and/or solubility.^28^

Solubility of cocrystals was increased with each coformer but remarkably improved (5 folds) with sodium acetate. This indicates the successful interaction of piroxicam with coformers and formation of cocrystals. The interaction between the pyridine and amide nitrogen atom of piroxicam and sodium acetate might have formed the cocrystal. The hydrogen bonding between pyridine and amide nitrogen of piroxicam and carboxylic acid leading to cocrystal formation was reported.^29^ Similar studies pertaining to solubility enhancement were reported with cocrystals of fluoxetine hydrochloride, niclosamide, meloxicam etc.^30-32^ Based on the results, piroxicam-sodium acetate cocrystal (called as piroxicam cocrystal in the following sections) was further characterized and used for the formulation of orodispersible tablets.

**Table 2:** Melting point and solubility of cocrystals

**Drug/Coformer**	**Melting point coformer**	**Cocrystal melting point (1:1)**	Solubility* (mg/ml) (1:1)	**Cocrystal melting point (1:2)**	Solubility* (mg/ml) (1:2)
Piroxicam	198-200		0.09769±0.32		
Piroxicam-sodium acetate	324	184-187	0.49166±0.61	189-191	0.30912±0.88
Piroxicam-saccharine sodium	277	181-183	0.11447±0.60	178-179	0.21515±0.49
Piroxicam-Urea	132-135	171-173	0.10727±0.65	175-177	0.13141±0.56
Piroxicam-Nicotinamide	125-131	162-165	0.10470±0.95	158-160	0.13532±0.77
Piroxicam-resorcinol	109 -112	185-187	0.10155±1.6	189-190	0.19292±0.23

*Average of three determinations Mean±SD

### Computational study 

The probable interaction between piroxicam and sodium acetate was studied by Schrödinger (Jaguar) software. The gas free energy of the piroxicam, sodium acetate and cocrystal was calculated. The piroxicam-sodium acetate complex showed least free energy (-1671.29) as compared to piroxicam (-1442.71) and sodium acetate (-228.49). The complex indicated greater stability owing to least free energy. Hence piroxicam may interact with sodium acetate via hydrogen bonding.

### IR spectroscopy

**Figure 1:**
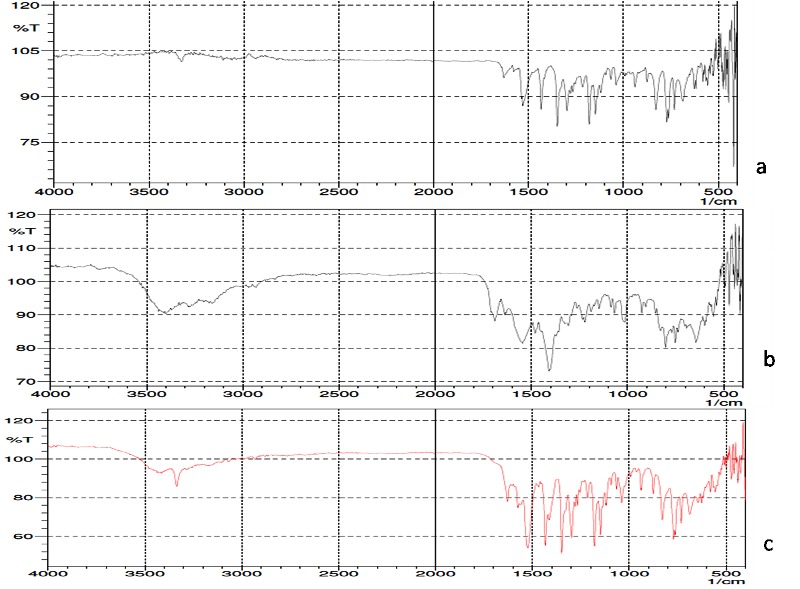
FTIR Spectra of a) Piroxciam b) Sodium acetate c) Piroxicam cocrystal

The IR spectrum for pure drug, coformer and cocrystal was recorded and shown in Figure 1. The principle bands were identified and associated changes were recorded. The IR spectrum of pure piroxicam shows the presence of the characteristic peaks which were recorded at 3334 cm^-1^ for NH stretching, SO2 stretching at 1147 cm^-1^, C-S stretching at 687 cm^-1^. The IR spectrum of sodium acetate revealed an absorption band at 3400 cm^-1^which can be assigned to O-H stretching. In addition C=H and C-O-C stretching bands were recorded at 1691 cm^-1^ and 1012 cm^-1^respectively. These spectra are in good agreement with the published data.^33^ The IR bands were significantly changed in the cocrystal in comparison to pure drug and coformer indicating interaction between drug and coformer. These alterations were manifested in the peaks corresponding to NH stretching which was observed at 3351 cm^-1^. This indicates cocrystal formation as peak shifted slightly, and became broader in the cocrystal. Many new peaks were observed in the cocrystal spectra supporting the formation of cocrystal. Similar changes in the IR spectrum of other drug like hydrochlorothiazide were reported and taken as indication of the cocrystal formation. Hence the changes recorded in the study can be taken as a signal of the cocrystal formation between the drug and coformers.^34^

### Differential scanning calorimetry

**Figure 2:**
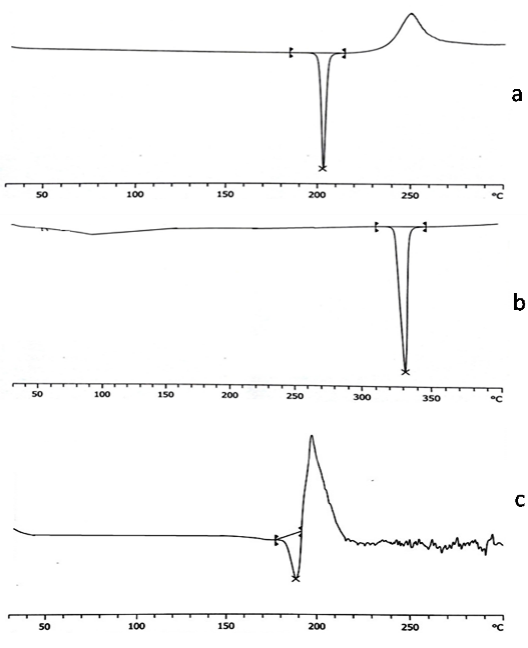
DSC thermogram of a) Piroxciam b) Sodium acetate c) Piroxicam cocrystal

Piroxicam, sodium acetate and piroxicam-sodium acetate cocrystal were characterized by DSC. The pure drug and coformer showed characteristic endothermic peak at 200.39 °C and 323.58 °C respectively corresponding to their melting point. Similar thermal behavior was reported for the drug.^35^The cocrystal showed substantial difference in the melting point (188.16°C) in comparison to pure drug (200.39°C) and coformer (323.58°C). Moreover, the peak onset for pure drug was obtained at 199.60 °C whereas 182.57 °C for cocrystal which indicates possibility of formation of the cocrystal. The peak corresponding to coformer fusion was not detected in the DSC of cocrystal that confirms the formation of cocrystal and thus absence of physical mixture. The change in the thermal properties were reported as evidence for the formation of cocrystal. Hence the present investigation denotes the formation of cocrystal.^36^ The DSC spectrum is shown in Figure 2. 

### Powder X-ray diffraction

**Figure 3:**
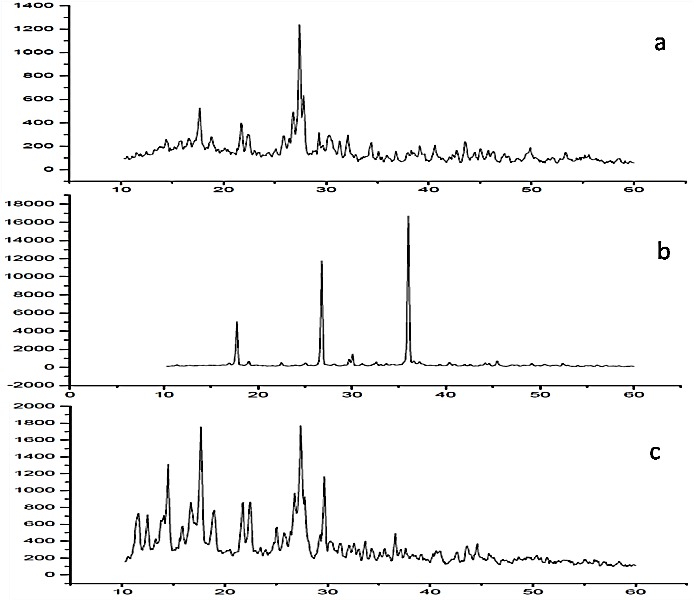
PXRD pattern of a) Piroxciam b) Sodium acetate c) Piroxicam cocrystal

The PXRD patterns for piroxicam, sodium actetate and cocrystal are shown in Figure 3. The materials in the powder state give distinctive peaks of varying intensities at certain positions. The diffractogram of the piroxicam showed characteristic diffraction peaks at different 2θ values (17.6, 17.7, 21.7, 27.4, 27.5, 27.8) indicating the crystalline nature. In addition diffraction peaks obtained for sodium acetate were 17.8, 26.7, 26.8, 35.9, 36, 36.1 2θ values. Similar diffraction pattern was reported in the previous investigations. The PXRD pattern of the cocrystal was distinguishable from its components and some additional diffraction peaks were appeared which did not exist in the pure drug or coformer. The additional diffraction peaks for cocrystal were obtained at 2θ values of 12.4, 12.5, 14.4, 14.5, 17.5, 17.6, 17.7, 17.8, 22.4, 22.5 27.3, 27.4, 27.5, 29.7, and 36.6. The appearance of new diffraction peaks in the diffractogram of cocrystal shows formation of new crystalline phase (cocrystal). The formation of cocrystals based on the PXRD pattern had been well documented, which showed new peaks that differ from the peaks corresponding to its input components.^37^

### Effect of pH on solubility of piroxicam

**Figure 4:**
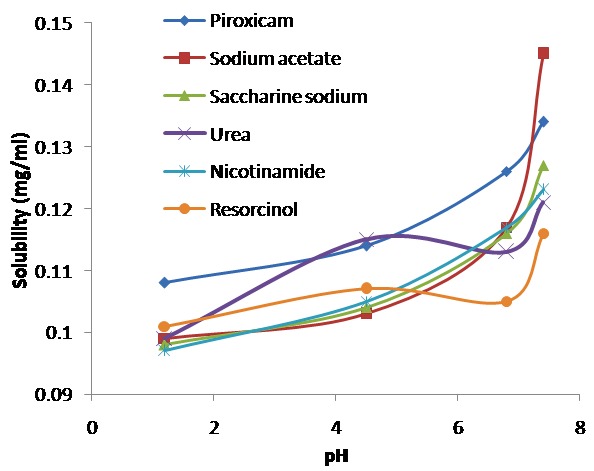
pH solubility profile of piroxicam and piroxicam cocrystal

The solubility of piroxicam was determined in the variety of buffers having pH 1to 10. The pH solubility profile was reported in Figure 4. The solubility of proxicam was different in the various buffers. The sigmoidal solubility curve was obtained. The solubility of piroxicam was not changed substantially till pH 5 but thereafter increased rapidly. The piroxicam is weakly acidic (pK_a1_ 1.86 and pK_a2_ 5.46) showing pH dependant ionization and solubility.

### Effect of pH on solubility of piroxicam cocrystal

The solubility of piroxicam cocrystal was estimated in the buffer solutions having pH 1.2, 4.5, 6.8 and 7.4. The pH solubility data was presented in the Figure 4. Cocrystal showed pKa dependant solubility and capricious behavior at different pH. The solubility of cocrystal was much greater at pH 7.4 as compared to piroxicam. This advocated the pairing of piroxicam cocrystals even at higher pH.^38^

### Powder dissolution study

**Figure 5:**
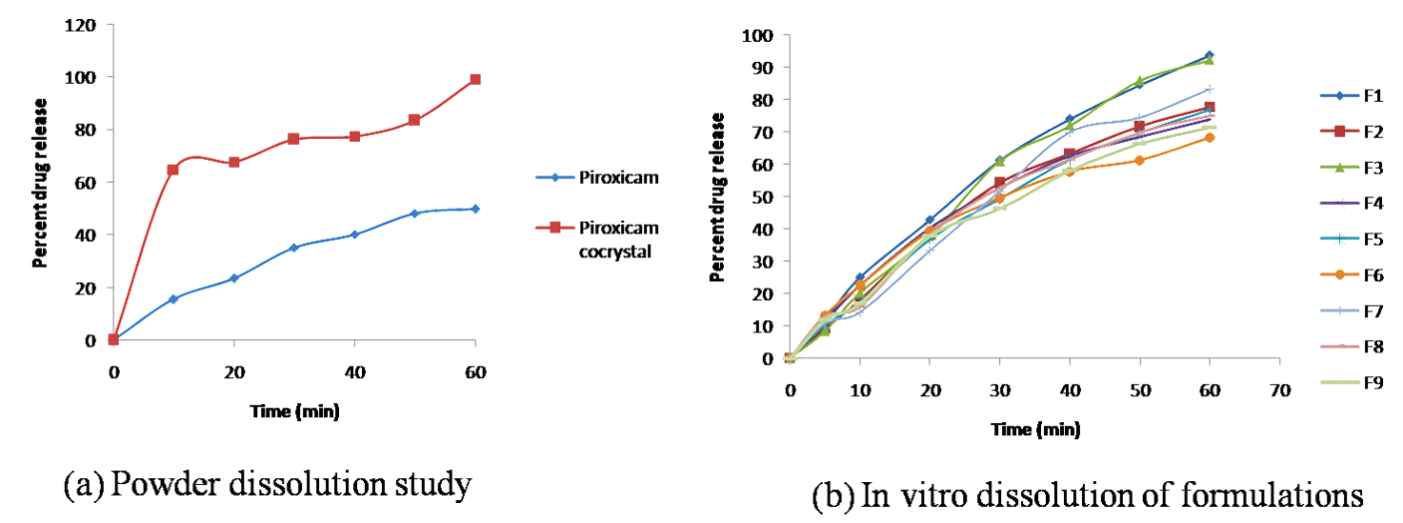
*In vitro* dissolution study

The dissolution rate plays crucial role in the bioavailability of drugs with poor solubility. The dissolution experiment was conducted on the pure drug and cocrystal. The dissolution profile of the pure drug and the prepared cocrystal are shown in Figure 5. The dissolution profile of pure drug indicates slow dissolution rate with only 15.62% of the drug being dissolved in the first 10 min. The total amount of drug dissolved in 60 min was 49.81% and calculated dissolution efficiency was only 29.8%. However cocrystal of the piroxicam resulted in significant increase in the dissolution rate. The amount of drug dissolved in first 10 min was 64.80% and the total amount dissolved was 99.10% with dissolution efficiency of 85.30%. This can indicate the weaker crystalline structure of the formed cocrystal as evident from higher dissolution rate. Moreover greater dissolution of piroxicam from cocrystal can be attributed to changed crystallinity pattern, size and shape and crystal habit of cocrystal that lead to enhanced solubility of cocrystal in the dissolution media. Cocrystallization had been well documented as a competent technique for dissolution enhancement.^39^ The similarity factor test denotes the dissolution of pure drug was dissimilar to the prepared cocrystal (F2 value 20%).

### Formulation of orodispersible tablets of piroxicam cocrystal by 3^2^ full factorial design

The present study was focused on formulation of orodispersible tablet of prepared piroxicam cocrystal. Preliminary studies were performed to optimize the concentration of superdisintegrant (crosscarmellose sodium) and binder (PVP K-30). The developed factorial formulations were subjected to evaluation of various precompression parameters and the results are depicted in Table 3. All the formulations exhibited good flow properties. The result of post compression parameters showed that, all the formulated tablets were of uniform weight with acceptable weight variation and thickness. Hardness of all formulations was maintained at 3.2-3.6 kg/cm^2^ and friability loss was between 0.72 to 0.86%. The hardness and friability studies revealed that the tablets possessed good mechanical resistance. The orodispersible tablets showed drug content between 98.04-99.48% which was within acceptable limits. The F1 batch was promising as it exhibited least disintegration time (29± 0.12 sec) and wetting time (21±0.58 sec), and maximum water absorption ratio (97.65±0.25%) (Table 3). The disintegration time was decreased with increasing concentration of superdisntegrant owing to sufficient swelling of tablet required for disintegration and wicking action of superdisintegrant.^40^

**Table 3:** Pre-compression and post compression parameters of designed formulations

**Precompression parameters**									
**Parameters**	**F1**	**F2**	**F3**	**F4**	**F5**	**F6**	**F7**	**F8**	**F9**
Bulk density (gm/cm^3^)	0.331±0.026	0.335±0.014	0.331±0.012	0.334±0.015	0.336±0.095	0.331±0.065	0.334±0.065	0.332±0.036	0.334±0.039
Tapped density (gm/cm^3^)	0.392±0.016	0.401±0.069	0.409±0.095	0.409±0.095	0.419±0.065	0.406±0.068	0.411±0.065	0.409±0.098	0.410±0.079
Hausner’s ratio	1.18±0.058	1.19±0.065	1.23±0.085	1.22±0.098	1.24±0.069	1.22±0.095	1.23±0.061	1.23±0.091	1.22±0.013
Compressibility index (%)	15.56±0.068	18.45±0.098	19.07±0.065	18.33±0.065	19.80±0.073	18.47±0.034	18.73±0.016	18.82±0.064	18.53±0.043
Angle of repose (θ)	29.92±0.032	26.96±0.065	29.54±0.021	30.92±0.064	30.96±0.015	31.31±0.024	28.25±0.054	29.51±0.064	31.27±0.079
**Post compression parameters**									
Weight Variation (mg)	200±1.3	201±1.9	200±1.5	201±0.9	201±1.3	199±1.3	198±1.1	202±2.6	200±1.6
Hardness (kg/cm^2^)	3.2±0.96	3.4±0.98	3.5±0.62	3.5±0.12	3.2±0.98	3.5±0.65	3.6±0.95	3.5±0.06	3.4±0.56
Thickness(mm)	4.22±0.7	4.18±0.8	4.15±0.4	4.16±0.8	4.22±0.2	4.17±0.2	4.14±0.1	4.16±0.5	4.19±0.3
Friability (%)	0.85±0.7	0.80±0.5	0.76±0.9	0.78±0.5	0.86±0.2	0.79±0.5	0.72±0.8	0.76±0.8	0.81±0.0
Disintegration time (sec)	29±0.12	41±0.95	32±0.65	36±0.97	33±0.58	32±0.65	42±0.15	34±0.85	40±0.25
Wetting time(sec)	21±0.58	30±0.35	22±0.36	26±0.86	23±0.58	29±0.35	32±0.76	29±0.68	24±0.25
Water absorption ratio(%)	97.65±0.25	81.35±0.98	88.59±0.5	84.62±0.36	89.74±0.49	88.16±0.36	79.39±0.84	89.06±0.62	83.39±0.67
Drug content (%)	99.48±0.2	98.04±0.5	99.18±0.8	99.08±0.9	99.01±0.4	99.02±0.5	99.44±0.5	98.46±0.7	98.48±0.3

Results are expressed as mean±standard deviation (n=3)

### In vitro drug release study

The study was aimed to evaluate the *in vitro* dissolution behavior of developed formulations. The drug release at 60 min was considered and depicted in Figure 5. The F1 batch showed maximum drug release (93.69±0.12%) although F3 batch exhibited comparable drug release. This might be due to lower concentration of binder and greater concentration of superdisintegrant. Depending on the entire evaluation parameters, F1 batch was selected as optimized formulation and subjected for stability study.^41^

### ANOVA study

Analysis of variance for dependent variables, disintegration time and percent drug release was performed. The coefficients X1(Crosscarmellose sodium) and X2 (PVP K-30) showed significant effect (p<0.05) on the selected responses.

### Response surface plots

**Figure 6:**
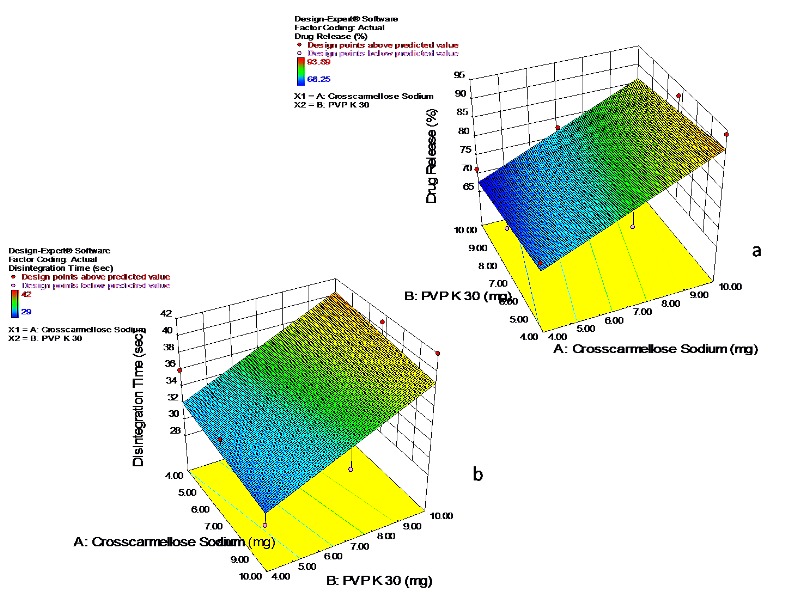
Response surface plots showing the effect of crosscarmellose sodium and PVP K-30

The response surface plots were generated for disintegration time and percent drug release and effect of independent variables, X1 and X2 was studied on the responses Figure 6.

The effect of formulation variables on disintegration time can be described by the model equation

Disintegration Time (sec) = +27.666-0.277 * X1 + 1.3868 * X2

The negative sign for coefficient X1 indicates increase in concentration of crosscarmellose sodium decreased the disintegration time and positive sign for X2 (PVP K-30) denotes as the concentration of X2 increased the disintegration time increased (R^2^=1) indicating good correlation between independent and dependant variables.

The parameter percent drug release can be described by model equation

% Drug release = + 62.03 + 3.11 * X1 – 0.6755 * X2

The positive sign for coefficient X1(crosscarmellose sodium) showed percent drug release increased with increase in concentration of X1 and negative sign for X2 (PVP K-30) indicates increased concentration of X2 decreases the percent drug release.

### Stability study

The optimized formulation F1 was subjected to stability study as per ICH guidelines. Color, odor, hardness, friability, drug content, disintegration time and percent drug release parameters were evaluated. The optimized formulation did not showed remarkable changes in these parameters (Table 4) and found stable at stability conditions.

**Table 4:** Stability study of optimized F1 formulation

**Formulation parameter**	**Ambient condition**	**30±2º/65±5% RH**	**40±2º/75±5% RH**
Color	white	white	white
Odor	No	No	No
Hardness (kg/cm^2^)	3.2±0.80	3.2±0.56	3.3±0.38
Friability (%)	0.84±0.8	0.86±0.34	0.85±0.46
Drug content (%)	99.28±0.3	99.05±0.17	99.14±0.21
Disintegration time(sec)	29±0.15	29±0.67	28±0.11
Percent drug release	93.49±0.11	93.63±0.14	93.39±0.28

Results are expressed as mean±standard deviation (n=3)

## Conclusion

The cocrystal of piroxicam was successfully prepared using sodium acetate as guest molecule to improve the solubility and dissolution. Dry grinding method allowed the formation of cocrystals. The cocrystal formation was confirmed by melting point alterations, DSC changes, shifts in Infra Red bands, changes in 2θ values in XRPD and mutually supported each others. The pH solubility profile of piroxicam and its cocrystals showed sigmoidal pattern. The Piroxicam cocrystals exhibited greater dissolution than the pure drug. The directly compressible orodipersible tablets of piroxicam cocrystal with shorter disintegration time, low friability, and greater drug release were developed by 3^2^ full factorial design. F1 formulation was found promising based on the evaluation parameters. The result indicated that, selected variables showed significant effect on the responses. Thus piroxicam cocrystals possessing modified physicochemical properties were obtained and successfully formulated as orodispersible tablets.

## Acknowledgments

Authors are thankful to Dr. Rupesh U. Shelke for helping in the computational study.

## Ethical Issues

Not applicable.

## Conflict of Interest

The authors declare no conflict of interests.
